# A group randomized trial of a complexity-based organizational intervention to improve risk factors for diabetes complications in primary care settings: study protocol

**DOI:** 10.1186/1748-5908-3-15

**Published:** 2008-03-05

**Authors:** Michael L Parchman, Jacqueline A Pugh, Steven D Culler, Polly H Noel, Nedal H Arar, Raquel L Romero, Raymond F Palmer

**Affiliations:** 1 VERDICT Health Services Research Center, South Texas Veterans Health Care System, San Antonio, TX, USA; 2 Department of Family & Community Medicine, University of Texas Health Science Center, San Antonio, TX, USA; 3 Department of Medicine, University of Texas Health Science Center, San Antonio, TX, USA; 4 Rollins School of Public Health, Emory University, Atlanta, GA, USA

## Abstract

**Background:**

Most patients with type 2 diabetes have suboptimal control of their glucose, blood pressure (BP), and lipids – three risk factors for diabetes complications. Although the chronic care model (CCM) provides a roadmap for improving these outcomes, developing theoretically sound implementation strategies that will work across diverse primary care settings has been challenging. One explanation for this difficulty may be that most strategies do not account for the complex adaptive system (CAS) characteristics of the primary care setting. A CAS is comprised of individuals who can learn, interconnect, self-organize, and interact with their environment in a way that demonstrates non-linear dynamic behavior. One implementation strategy that may be used to leverage these properties is practice facilitation (PF). PF creates time for learning and reflection by members of the team in each clinic, improves their communication, and promotes an individualized approach to implement a strategy to improve patient outcomes.

**Specific objectives:**

The specific objectives of this protocol are to: evaluate the effectiveness and sustainability of PF to improve risk factor control in patients with type 2 diabetes across a variety of primary care settings; assess the implementation of the CCM in response to the intervention; examine the relationship between communication within the practice team and the implementation of the CCM; and determine the cost of the intervention both from the perspective of the organization conducting the PF intervention and from the perspective of the primary care practice.

**Intervention:**

The study will be a group randomized trial conducted in 40 primary care clinics. Data will be collected on all clinics, with 60 patients in each clinic, using a multi-method assessment process at baseline, 12, and 24 months. The intervention, PF, will consist of a series of practice improvement team meetings led by trained facilitators over 12 months. Primary hypotheses will be tested with 12-month outcome data. Sustainability of the intervention will be tested using 24 month data. Insights gained will be included in a delayed intervention conducted in control practices and evaluated in a pre-post design.

**Primary and secondary outcomes:**

To test hypotheses, the unit of randomization will be the clinic. The unit of analysis will be the repeated measure of each risk factor for each patient, nested within the clinic. The repeated measure of glycosylated hemoglobin A1c will be the primary outcome, with BP and Low Density Lipoprotein (LDL) cholesterol as secondary outcomes. To study change in risk factor level, a hierarchical or random effect model will be used to account for the nesting of repeated measurement of risk factor within patients and patients within clinics.

This protocol follows the CONSORT guidelines and is registered per ICMJE guidelines:

**Clinical Trial Registration Number:**

NCT00482768

## Background

Although tight control of glucose (A1c), blood pressure (BP), and lipids can prevent complications from type 2 diabetes [1-4], a substantial proportion of patients with type 2 diabetes seen in primary care settings have poor control of one or more of these risk factors [5-7]. According to the Chronic Care model (CCM), patient outcomes such as good control of these risk factors should be associated with the presence of one or more of the following elements within the health care organization: organizational leadership, self-management support, delivery system design, decision support, clinical information systems, and community linkages [8,9]. Barriers to implementing the CCM elements in primary care include a lack of motivation of key stakeholders, no external motivators for change, a paucity of resources, and no perceived opportunities to implement change [10,11].

### Assumptions behind organizational interventions

When we design or use organizational interventions to improve patient outcomes, we make assumptions about the nature of the system we are targeting. Many prior attempts to design interventions for primary care settings were based on a mechanistic approach: each practice or clinic has a broken or sub-standard 'part' that needs to be isolated and 'fixed.' These approaches have consistently provided disappointing results [12]. A recent review of such efforts revealed only a 9% improvement across all clinical practice guideline implementation studies [13]. In a review of strategies to improve glycemic control, the Agency for Healthcare Research and Quality's funded Evidence Based Practice Center identified 27 studies that employed organizational interventions [14]. They concluded: '...organizational change as a broad category had little impact on glycemic control....' What do we know about primary care teams that would inform the development of a more effective intervention to overcome these barriers in primary care settings?

### Primary care clinics are complex adaptive systems

Recent conceptualizations of the health care system of the 21st century call for recognition of the complex, adaptive nature of primary care settings [15]. Conceptualizing primary care practices as complex adaptive systems (CAS) facilitates understanding their current state, health systems context, and potential for change in response to interventions [10,16]. A CAS is a collection of individuals (*e.g.*, clinicians, staff, administrators, and patients) whose actions are interconnected such that one person's action changes the context for other individuals in the system [17]. Although these individuals can behave in unpredictable ways, they usually act according to a set of stated and unstated simple rules [18,19]. Agents in a CAS tend to repeat patterns of activities that serve their particular values and motivations, making transformation difficult because changes are met by pressures to maintain the status quo [20]. CASs have multiple feedback loops by which agents organize and reorganize based upon nonlinear interactions [21].

The ability of a CAS to adapt in a manner that allows for change or improvement can be enhanced by improving the quality and quantity (bandwidth) of communication among agents [17-19]. In a study of organizational features that support innovation in primary care practices, a key feature was an increase or improvement in communication and participation among people at all levels of the practice [11]. Another example of the importance of communication can be found in studies regarding implementation of Electronic Medical Records. (EMRs) Although EMRs are seen as potentially effective strategies to improve quality and outcome of care, a failure to resolve communication problems between agents in the system, rather than a failure to resolve information technology problems, is a major cause of failed implementation [22]. The central question for any translational research effort in primary care settings is: how can we leverage the properties of a primary care CAS to develop sustainable interventions that will improve patient outcomes across a wide diversity of primary care settings? One such approach is practice facilitation.

### CAS theory and practice facilitation

Practice Facilitation (PF) is an intervention that exploits these CAS properties and overcomes the aforementioned barriers [23,24]. PF occurs when a trained facilitator meets with staff and clinicians in each practice over several months to assist the team in addressing an issue, such as improving risk factors for diabetes complications. The facilitation is guided by insights from an in-depth multi-method assessment process in each practice prior to the facilitation intervention. Facilitation meetings create time for learning and reflection by members of the team. This in turn helps the practice team improve their communication so that they can adopt and implement a strategy to improve patient care. It has proven effective in the primary care setting for improving quality of care processes for rates of colorectal cancer screening [24], health habit counseling [25], and the quality of asthma care for children [26]. Although the effectiveness of in primary care settings has been demonstrated for process measures of quality, its effectiveness in improving clinical outcomes such as A1c, BP, or lipids for patients with type 2 diabetes has not been tested. In addition, little is known about the process through which PF might improve patient outcomes.

The purpose of the proposed group randomized controlled trial is three-fold: 1) to improve risk factors for type 2 diabetes complications across a diversity of primary care clinics through PF; 2) to examine the relationship between implementation of the CCM and communication among staff and clinicians; and 3) to advance the science of translational research in primary care settings by examining the sustainability of the intervention. The specific objectives are to:

1. Evaluate the effectiveness and sustainability of PF to improve risk factors for type 2 diabetes complications across a variety of primary care settings.

Hypothesis 1a: Patients within intervention practices will have lower A1c, blood pressure and lipid levels than those in control practices.

Hypothesis 1b: This improvement will be sustained over the 12-month period after withdrawal of the PF intervention.

2. Assess the implementation of the CCM in response to the intervention.

Hypothesis 2a: Compared to control practices, practices in the intervention group will improve their delivery of CCM elements.

Hypothesis 2b: This change will be sustained 12 months after the intervention is withdrawn.

Hypothesis 2c: Implementation of the CCM elements will be associated with risk factor control, but this association will be stronger in the intervention clinics.

3. Examine the relationship between communication within the practice team and the presence of the CCM elements.

Hypothesis 3a: Communication among staff and clinicians within intervention clinics will improve compared to control clinics.

Hypothesis 3b: This improvement in communication will be sustained 12 months after the intervention is withdrawn.

Hypothesis 3c: Communication among staff and clinicians will be associated with a change in the presence of the CCM elements, but this association will be stronger in intervention clinics.

4. Determine: the cost of the intervention from the perspective of the organization providing the PF intervention activities; the net cost (revenue minus cost of services and intervention) from the perspective of a primary care practice; and the costs per change in risk factor from each perspective. (This objective is descriptive. No hypotheses are postulated.)

## Methods

### Study setting and subjects

The subjects of this study will be 40 primary care clinics known as 'practices' in a large practice-based research network, the South Texas Ambulatory Research Network. Inclusion criteria for the study are: 1) the practice must have seen at least 60 patients with type 2 diabetes in the past year (in order to insure our sample size of 60 patients per practice); 2) they must be willing and able to use their billing records to identify these patients; and 3) representative members, clinicians, and office staff in the practice must agree to meet with the practice facilitator on a regular basis for one-hour team meetings over 12 months. Exclusion criteria are: 1) multi-specialty practices; 2) practice owned by a large vertically integrated health care system; and 3) practices with five or more physicians.

### Design

This will be a cluster-randomized trial with 20 clinics in the intervention arm and 20 in the control arm. (see Additional File 1) Because the intervention will be implemented in groups of five clinics at three-month intervals, a block randomization to intervention or control groups will be done so that there are four blocks with ten clinics in each block. The randomization scheme will be computer-generated in SPSS 15.0 by the study statistician. Neither the investigators nor the subjects will be blinded. The final chart abstraction for primary and secondary outcomes will be done by a trained abstractor who is blinded to allocation.

### Data collection

To obtain the dependent and independent variable necessary to accomplish specific aims one through three, clinician and staff surveys will be administered and medical record abstracted in each clinic. Site visits will be conducted in all 40 clinics and will be conducted three times: at baseline after enrollment but prior to randomization, and 12 and 24 months after starting the initial facilitation intervention in each clinic. During each site visit, clinicians and staff will complete surveys to measure the presence of the elements of the CCM as well as communication among clinicians and staff (see description of outcomes below).

The second method of data collection is a blinded medical record abstraction for the primary outcomes: A1c, BP, and lipid levels. This will be accomplished by a trained chart auditor who is blinded to assignment of clinics to intervention or control groups. The abstraction will take place at baseline, after the conclusion of the delayed intervention in the control clinics and 12 months after the end of the intervention in the initial intervention clinics.

For the fourth specific aim, a project accounting system will be developed to allocate all project expenses to a set of cost categories (cost pools) to assess the cost from the perspective of the organization conducting the intervention. Definitions and rules for assigning expenses into cost pools will be developed by the PI and project director in the first three months of the project. The second goal of specific aim four is to estimate the direct variable cost of conducting PF in the typical primary care facility. Net cost to the practice of implementing the intervention is: Revenues – (service cost plus cost resulting from intervention). Revenue will be tracked from billing data downloaded from each practice during each of the three site visits to track utilization and charges for all patients with type 2 diabetes in each practice for the 12 months prior to intervention, and at the end of 12 month period following the first facilitation visit. The methods used to collect cost data include meeting with the office manager at each practice during each site visit, and the collection of detailed field notes by the facilitators during direct observation in each practice. These data will be used to estimate the fixed and incremental cost of all resources used by the practice to implement the new strategy. We anticipate that these new resources will vary from practice to practice depending on the number of strategies implemented by the practice as a result of the intervention.

### Outcomes

Patient-level outcomes will be measured by collecting data on a random sample of medical records on 60 patients within each clinic at the conclusion of the delayed intervention. All dates and values of A1c, BP, and lipids for the prior 12 months will be collected at baseline and for the intervening time period during the final chart abstraction, for a total of 36 months of values. The random sample of medical records of patients with type 2 diabetes will be selected from a list of all patients with type 2 diabetes seen within each clinic over the prior 12 months generated from each practice's billing system.

Practice-level outcomes will be measured by physician and staff surveys administered three times: at baseline, at the conclusion of the 12-month intervention, and 12 months later. The extent to which the care delivered in each clinic is consistent with the elements of the CCM will be measured with the Assessment of Chronic Illness Care survey (ACIC) [27]. The ACIC measures the presence of the six elements of the CCM. Each item is scored on a 0 to 11 scale and provides sub-scale scores for each of the six CCM components as well as a total score. The validity of the instrument is supported by the findings of a study of an intervention for diabetes and congestive heart failure: all six sub-scales were responsive to process of care improvement [27].

Communication among staff and clinicians will be measured with a survey developed by Shortell and colleagues that was previously validated in health care settings [28]. This instrument captures three aspects of organizational communication: openness [29], timeliness [28] and accuracy [30]. These aspects as measured by this specific instrument have been shown to influence the ability or willingness of health care workers to develop relationships that increase the number and quality of interconnections and information flow, contributing to better self-organization and outcomes [20].

### Baseline practice assessment

Prior to the first practice team meeting, the facilitators will conduct a one-week detailed assessment in each of the 20 intervention practices [31-33]. This data will be used to prepare an initial practice report that will be used in the first step of the intervention. The data from the assessment will be used to locate potential change points for improving practice change capacity and diabetes service delivery.

The primary data of the assessment will be dictated field notes from observations of the practice environment and clinical encounters. A detailed template will be used as a reminder to the facilitator of topics to be included in the field notes. Observational field notes [34] will be supplemented with collection and review of existing practice documents, including medical charts, flow sheets, patient schedules, personnel lists, mission statements, office protocols, and annual reports. Key informant interviews will be conducted to develop a more detailed understanding of clinician, staff, and patient perception of their goals and performance [35]. Separately, the facilitator will gather data using a standardized medical record review form to obtain performance data on risk factor control (A1c, BP and Lipids) for 60 patients with diabetes in each clinic (see outcomes below). Clinician and staff survey data collected during the initial site visit (see outcomes below), as well as practice characteristics will also be incorporated into the assessment. All qualitative data will be recorded, transcribed, and entered into a text management software program, and an in-depth analyses will be done to guide the subsequent facilitation intervention.

### The intervention: PF and the facilitator's toolbox

Each practice facilitator will be assigned ten intervention practices, and will meet with team members in their assigned practices initially once every other week for three to six months, and then monthly over a period of 12 months. During each meeting the facilitator will assist the team in tailoring and implementing a strategy to improve risk factors that emerges out of the discussion of five strategies from the 'toolbox.' (see below) The practice facilitator will remain available to each practice for *ad hoc *consultation between team meetings during this 12-month period. Each meeting will last one hour.

The PF intervention will follow the principles described by Crabtree, Miller and Stange in their series of studies to improve health habit and cancer screening activities in primary care practice settings [23,25]. One of the key attributes of PF is creating protected time and space for members of the practice to reflect on a given issue and tailor evidence-based strategies to improve diabetes care outcomes in a manner that is consistent with their resources, organizational culture and values, and history. The emphasis will be on a common goal: improving risk factors for diabetes complications.

### Facilitation toolbox

Each facilitator will have resources and material on five strategies to improve diabetes outcomes in a 'toolbox' of ideas and will share these with the members of each practice during the first few sessions. Examination of the literature suggests that there is some evidence for potential effectiveness of five strategies: 1) implementation of a diabetes registry [36,37]; 2) point-of-care testing for A1c and/or lipids [38,39]; 3) group clinic visits [40,41]; 4) clinical reminders and decisions support [42,43]; and 5) patient activation [44]; [45]. Practices will not be limited to these five strategies. A discussion of each of these tools in the 'toolbox' will occur as an initial step in the facilitation intervention. The purpose of this discussion is to stimulate the practice team to adapt and implement one or more of the five strategies or to develop their own innovative strategy to improve risk factors or both. This is consistent with current theory regarding primary care practices as complex adaptive systems [16,17].

### The delayed intervention

Insights gained during the initial 12-month intervention will be used to design **a **refined and enhanced delayed facilitation intervention in the practices initially randomized to the control group. This design allows initial learning about intervention techniques to be rapidly tested in the delayed intervention practices after they have served as controls. Importantly, the design also provides an incentive for the control practices to participate, because instead of just providing control data, they later receive a refined intervention. It is also important to note that institutional review boards are increasingly questioning the ethics of not offering control subjects and settings some benefit from participation in an RCT. This delayed intervention will help address those concerns. The delayed intervention will be similar process to the PF described above. However, the knowledge and skills acquired in the first 20 practices will be used to refine and enhance both the evidence-based strategies in the facilitator toolbox. This delayed intervention will be evaluated in a pre-post design.

### Sample size and analysis

To test hypotheses for specific aim one, the unit of randomization will be the clinic and the unit of analysis will be the repeated measure of each risk factor for each patient, nested within the clinic. The outcome will be the level of control of each risk factor. We will examine A1c as our primary outcome, with BP and LDL-cholesterol as secondary outcomes. To study change in risk factor level, a hierarchical or random effect model will be used to account for the nesting of repeated measurement of risk factor within patients and patients within clinics [46,47].

The power calculation is derived for the planned cluster randomized design with 20 clinic in each treatment arm and 40 patients each clinic under the hierarchical linear analysis plan (random effects models), and the significance level set at 0.05. For the first specific aim, our primary outcome is A1c and the power estimates are calculated based on the interclass correlation coefficient (ICC) for A1c obtained from a preliminary study of 20 practices [48]. That value is 0.113. For the first specific aim, if the mean decrease in A1c due to intervention is 0.7 or greater, then the power for detecting the intervention effect on A1c is 0.80.

For specific aims two and three, the ACIC score (for specific aim two) and the communication score (for specific aim three) measured repeatedly at the staff and clinician level will be the outcome of primary interest, as it will reflect the presence of elements of the CCM. Due to a similar distributional nature of the ACIC or communication score and risk factors (ACIC and communication scores are measured repeatedly at three time points at the staff level nested within each clinic and are continuous), the three-level random effects model as proposed for specific aim one is appropriate. Sample size and power calculations for these aims are similar to those for the first aim. For the second aim, the ICC of the ACIC score is 0.12 with a standard error of 2.15, resulting in a power of 0.94 to detect a change in ACIC score of at least 1.5 in response to the intervention. For the third specific aim, the ICC for communication scores is 0.33 and the associated standard error is 9.46, thus the power for detecting the intervention effect on communication score is no less than 0.81 if the mean communication score in the intervention group is seven points or greater compared to that for the control group.

For specific aim four, the difference in revenues generated by the practice for all services provided to patients with diabetes for the 12 months prior to the intervention compared to the 12 months during the facilitation intervention will be determined. Second, the cost of providing services to each patient with diabetes in each practice will be estimated using CPT codes for services delivered at that CPT codes Relative Value Units from MedPar files [49]. Finally, the incremental cost of implementing the strategy to improve risk factor control in each practice will be estimated.

### Ethics

This protocol received human subjects protection approval from the Institutional Review Board at the University of Texas Health Science Center at San Antonio on 19 March 2007. (IRB protocol HSC20070546H)

## Competing interests

The author(s) declare that they have no competing interests.

## Authors' contributions

MLP conceived and developed the study, drafted the study protocol, and leads the implementation. JAP, PHN and SDC helped to draft both the study protocol and this manuscript. RLR coordinates the ongoing study, collected pilot data, and helped to draft the manuscript. NHA and RFP are members of the Study Steering Group, and have contributed to the development of the protocol. All authors read and approved the final manuscript.

## Supplementary Material

Additional file 1 Study Overview Click here for file
