# Supplementary material for: A group randomized trial of a complexity-based organizational intervention to improve risk factors for diabetes complications in primary care settings: study protocol
Source: Implement Sci. 2008 Mar 5;3:15. doi: 10.1186/1748-5908-3-15 (PMC2291070; doi:10.1186/1748-5908-3-15)
Supplement: Additional file 1 — Study Overview [file 1748-5908-3-15-S1.doc]

Enroll Practices, Site Visit-1 & Practice Assessment

Intervention Practices

Control Practices

Facilitation Intervention

No intervention

Site Visit-2

Site Visit-2

No Facilitation

Delayed Facilitation

Intervention

Site Visit-3

Site Visit-3

**Appendix A: Overview of Study Design**

Randomization

RCT

Pre-Post Design

Final Medical Record Abstraction

**Timeline**

Baseline

12 months

24 months

**Study Design**
